# Type 2 Low Biomarker Stability and Exacerbations in Severe Uncontrolled Asthma

**DOI:** 10.3390/biom13071118

**Published:** 2023-07-13

**Authors:** Arja Viinanen, Juhani Aakko, Mariann I. Lassenius, Gunilla Telg, Kaisa Nieminen, Saara Kaijala, Lauri Lehtimäki, Hannu Kankaanranta

**Affiliations:** 1 Division of Medicine, Department of Pulmonary Diseases, Turku University Hospital, 20014 Turku, Finland; 2 Department of Pulmonary Diseases and Clinical Allergology, University of Turku, 20014 Turku, Finland; 3 Medaffcon Oy, 02130 Espoo, Finlandsaara.kaijala@medaffcon.fi (S.K.); 4 AstraZeneca, 51 85 Stockholm, Sweden; 5 AstraZeneca, 02150 Espoo, Finland; 6 Allergy Centre, Tampere University Hospital, Faculty of Medicine and Health Technology, Tampere University, 33014 Tampere, Finland; 7 Krefting Research Centre, Department of Internal Medicine and Clinical Nutrition, Institute of Medicine, Sahlgrenska Academy, University of Gothenburg, 417 56 Gothenburg, Sweden; 8 Faculty of Medicine and Health Technology, Tampere University, 33014 Tampere, Finland; 9 Department of Respiratory Medicine, Seinäjoki Central Hospital, 60220 Seinäjoki, Finland

**Keywords:** severe uncontrolled asthma, type 2 low, blood eosinophils, fractional exhaled nitric oxide, exacerbations, adults

## Abstract

We investigated the stability of T2 low status, based on low levels of T2 biomarkers, and exacerbation rates in T2 low and non-T2 low asthma from clinical retrospective data of severe uncontrolled asthma patients. Knowledge of the T2 low biomarker profile is sparse and biomarker stability is uncharted. Secondary care patients with severe uncontrolled asthma and at least two blood eosinophil counts (BEC) and fractional exhaled nitric oxide (FeNO) measured for determination of type 2 inflammation status were evaluated from a follow-up period of 4 years. Patients were stratified into four groups: T2 low_150_ (*n* = 31; BEC < 150 cells/µL and FeNO < 25 ppb), non-T2 low_150_ (*n* = 138; BEC > 150 cells/µL and/or FeNO > 25 ppb), T2 low_300_ (*n* = 66; BEC < 300 cells/µL and FeNO < 25 ppb), and non-T2 low_300_ (*n* = 103; BEC > 300 cells/µL and/or FeNO > 25 ppb). Exacerbation rates requiring hospital care, stability of biomarker status, and cumulative OCS and ICS doses were assessed during follow-up. Among patients with severe uncontrolled asthma, 18% (*n* = 31) were identified as T2 low_150_, and 39% (*n* = 66) as T2 low_300_. In these groups, the low biomarker profile was stable in 55% (*n* = 11) and 72% (*n* = 33) of patients with follow-up measures. Exacerbation rates were different between the T2 low and non-T2 low groups: 19.7 [95% CI: 4.3–45.6] in T2 low_150_ vs. 8.4 [4.7–13.0] in non-T2 low_150_ per 100 patient-years. BEC and FeNO are useful biomarkers in identifying T2 low severe uncontrolled asthma, showing a stable follow-up biomarker profile in up to 72% of patients. Repeated monitoring of these biomarkers is essential in identifying and treating patients with T2 low asthma.

## 1. Introduction

Two major asthma endotypes have been described based on the presence of type 2 inflammation (T2): type 2 high and low asthma. Key mediators of type 2 inflammation, namely interleukin (IL)-4, -5, and -13, can be produced by type 2 T-helper cells (Th2) or type 2 innate lymphoid cells, contributing to the type 2 high signature, which is the most well-defined endotype with eosinophilic inflammation [1]. Type 2 low asthma is characterized by the absence of signs and markers of type 2 inflammation and has been proposed to present with either neutrophilic or paucigranulocytic (absence of granulocytes) inflammation. Type 2 low inflammation tends to be more resistant to inhaled corticosteroids [2], suggesting an unmet need for appropriate medication [3].

Interleukin 5 is a key driver of synthesis and activation of eosinophils, the predominant effector cell type in type 2 high asthma. In parallel, blood eosinophil count (BEC) has gained widespread acceptance as a surrogate of airway eosinophilia [4]. Type 2 high airway inflammation also drives the transcription of inducible nitric oxide (NO), thus increasing NO production. Fractional exhaled nitric oxide (FeNO) predominantly indicates IL-13 activity, with higher FeNO concentrations suggesting type 2 airway inflammation and steroid responsiveness [5]. In a recent consensus statement, a cut off of 25 ppb for FeNO was proposed for defining type 2 inflammation, whereas the BEC cut-offs were 300 cells/µL and 150 cells/µL in combination with other features of probable type 2 inflammation [6]. Type 2 cytokines, especially IL-4, also promote the production of immunoglobulin E (IgE) from B lymphocytes, increasing the expression of IgE receptors on B lymphocytes and macrophages while directing class switching of naïve CD4 T-helper lymphocytes, further enhancing inflammation [4]. Low BEC, FeNO, and IgE may help in identifying patients with type 2 low endotype, lacking the typical inflammatory profile in type 2 high asthma.

Currently, there is a lack of understanding on what proportion of patients with severe uncontrolled asthma present with type 2 low status and on the characteristics of patients identified as having type 2 low asthma based on the biomarkers BEC and FeNO [7,8]. Further, different BEC cut off values (150 and 300 cells/µL) have been used in different studies to mark eosinophilic disease [9,10,11]. Moreover, there is a paucity in information on how the type 2 low biomarker profile is sustained over time and the focus has been on type 2 high asthma [12]. Understanding the heterogeneity of asthma phenotypes and endotypes and achieving a precise characterization of type 2 low asthma is important to guide treatment decisions [13,14,15]. Hence, the aim of the study was to investigate different biomarker profiles, widely available in routine clinical practice and also used to guide clinical treatment, in defining type 2 low asthma and to assess the stability of biomarker status over time. Further, we investigated exacerbations requiring hospital care stratified by type 2 status, bringing novel information on differences and similarities between type 2 low and non-type 2 low patients. We focused on patients with severe uncontrolled asthma stratified per type 2 status, as these patients have a significant unmet medical need.

## 2. Materials and Methods

### 2.1. Patients

All patients with an asthma diagnosis (J45.x, J46.x) and a visit to the specialist pulmonary department at Turku University Hospital between 1 January 2012–31 December 2017 were included in the analyses. In this prevalent patient setting, index was defined as 1 January 2018, and patients were followed until death or 31 December 2021, see Figure S1. Data from Turku University Hospital Auria data lake included all specialty care contacts, diagnoses, procedures, date of death, spirometry data, FeNO concentrations, asthma control test results, and other laboratory measurements including BEC and total IgE. Data on drug purchases of ATC classes R* (respiratory system), A10* (diabetes), C* (cardiovascular system), H02* (systemically used corticosteroids), J* (anti-infectives for systemic use), N06A* (antidepressants), and M05* (drugs for treatment of bone diseases) were obtained from the Social Insurance Institution of Finland (SII) and were linked to Auria data (permission number THL/2385/14.02.00/2021). The study permission was granted by the central permission authority in Finland, Findata. No ethical approval or consent was required due to the retrospective registry-based design of the study, in accordance with the Act on the Secondary Use of Social and Health Data, Finlex 522/2019 [16].

### 2.2. Asthma Severity and Uncontrolled Asthma

Drug purchase data from the first ICS purchase onwards during baseline (1 January 2012–31 December 2017) were used to assess asthma severity. The average daily use of fluticasone propionate (FP) equivalent was calculated based on a sliding window of four consecutive ICS purchases, where the average use was defined as the total µg of FP equivalent of the four consecutive purchases divided by the number of days between the first and fifth consecutive purchase. Asthma patients with a daily use of ≥800 µg of FP equivalent within this time window and with at least one other controller (Leukotriene Receptor Antagonists (LTRA), Long-Acting Beta-Agonist (LABA), Long-Acting Muscarinic Antagonists (LAMA), or biologic asthma medication) during the same time window of ICS use were identified as patients with severe asthma. Per ERS/ATS guidelines, severe asthma is defined based on the use of at least 1000 µg/day of FP [17]; we allowed for 80% adherence and hence ≥800 µg of FP equivalent per day was required. None of the patients had been treated with a biological asthma drug. Uncontrolled asthma was defined as asthma control test (ACT) score <20 or having an emergency room (ER) visit or hospitalization for asthma with acute asthma (ICD-10: J46), or asthma as a main diagnosis (J45.x), or a respiratory infection as a main diagnosis + asthma (J45.x) as a side diagnosis.

### 2.3. Type 2 Low Status

To explore the type 2 low biomarker status, two different blood eosinophil count (BEC) cut offs (150 and 300 cells/µL) were utilized in the analyses. At least two measurements of BEC persistently below the threshold of 150 or 300 cells/µL and one or more FeNO measurement consistently below 25 ppb during baseline were required to fulfil the type 2 low criteria. Based on these criteria, patients were stratified into those meeting the BEC < 150 cells/µL + FeNO < 25 ppb, called T2 low_150_. If a patient only met one of the T2 low criteria based on BEC and FeNO or displayed consistently higher values during baseline (BEC ≥ 150 cells/µL and/or FeNO ≥ 25 ppb), they were called non-T2 low_150_. For BEC <300 cells/µL + FeNO <25 ppb cut off, the corresponding groups were named T2 low_300_ and non-T2 low_300_ (BEC ≥ 300 cells/µL and/or FeNO ≥ 25 ppb during baseline). Patients from the T2low_150_ group were included in the T2low_300_ group.

Among those defined as T2 low, the overlap of the applied BEC and FeNO criteria are presented in Figure S2.

### 2.4. Stability of the Type 2 Low Biomarker Status and Exacerbations

During follow-up, the stability of the T2 low_150_ and T2 low_300_ status was evaluated based on the continued measures of BEC <150 or <300 cells/µL and FeNO <25 ppb [6]. If the threshold was exceeded during follow-up, the status was considered non-stable. The type 2 low biomarker stability during follow-up was assessed and visualized with swimmer plots. The proportion of patients with a stable biomarker profile of either BEC and/or FeNO was reported in those with at least one measure available during follow-up, excluding patients with no consecutive measurements from the calculation.

Exacerbations were defined as hospitalizations or ER visits with acute asthma (ICD-10 code J46) as a main or side diagnosis, asthma as a main diagnosis (J45.x), or respiratory infections (J00.x-J22.x) as a main diagnosis and asthma (J45.x) as a side diagnosis. Event rates for the exacerbations were computed by dividing the number of events during the follow-up by the total follow-up time in years. The 95% confidence intervals for the event rates were obtained with bootstrapping. Mean cumulative count for the exacerbations was estimated by a mean cumulative function. The analyses were stratified by the subgroups.

### 2.5. Analyses

Where applicable, differences between populations were tested using relevant statistical tests (two-sided T-test for normally distributed continuous variables, Kruskal–Wallis test for non-normally distributed continuous variables, and Chi-squared test for categorical variables).

The mean cumulative dose of OCS and ICS during follow-up was estimated by a mean cumulative function. The results were plotted with 95% confidence intervals over time. *p*-values less than 0.05 were considered statistically significant. All analyses were performed using R-4.0.3 [18].

## 3. Results

### 3.1. Cohort Formation and Different BEC Cut Offs

**Figure 1:**
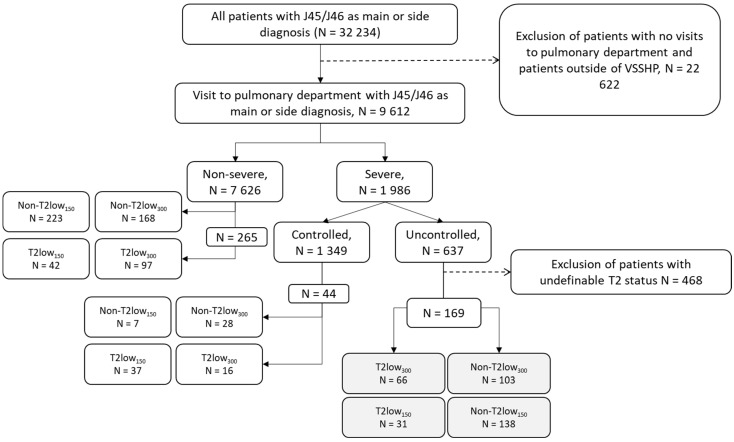
Cohort formation of patients with severe uncontrolled asthma with T2 low_300_ and T2 low_150_ criteria. Non-T2 low—those not meeting the T2 low criteria.

Overall, 9612 patients were included in the analyses; the distribution of T2 low status among non-severe and severe patients is shown in Figure 1. Severe asthma was identified in 1986 (20.7%) patients. Of these, 32% (*n* = 637) had uncontrolled asthma, and 169 patients had biomarker data to be assessed for type 2 status. Among the patients with severe uncontrolled asthma, the focus of this study, the overlap of the BEC and FeNO criteria for both type 2 low cut offs, are presented in Figure S2. Of the severe uncontrolled asthma patients with biomarker data available for classification, 18% (*n* = 31) were classified as T2 low_150_, and 39% (*n* = 66) as T2 low_300_.

### 3.2. Clinical Characteristics

The clinical characteristics of patients with T2 low and non-T2 low are presented in Table 1. Severe uncontrolled T2 low patients were on average younger and more often females than corresponding patients with non-T2 low asthma, seen across both BEC 150 and 300 cut offs. Other markers and signs of type 2 inflammation were also significantly lower in T2 low patients, including maximal total IgE [24 (10, 77) vs. 222 (53, 427) kU/l, *p* < 0.01 in T2low_300_ vs. non-T2 low_300_] and nasal polyps that were absent in T2 low patients vs. observed in 10–14% of non-T2low patients. Neutrophil counts and respiratory antibiotic purchases were similar between groups during baseline.

The ACT indicated poor asthma control with a median score of 11–12 points in both T2 low and non-T2 low patients across both BEC categories. FEV_1_ and FEV_1_/FVC values were slightly higher in T2 low patients vs. non-T2 low. The median daily OCS and ICS doses based on medication purchases during baseline were similar between T2 low_150_ and non-T2 low_150_ as well as T2 low_300_ and non-T2low_300_, Table 1.

### 3.3. Stability of Type 2 Low Biomarker Status during Follow-Up

**Figure 2:**
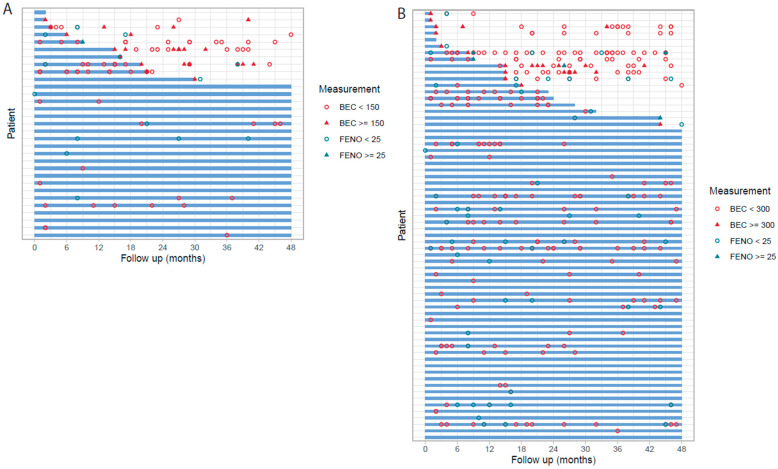
Biomarker stability among severe uncontrolled T2 low asthma patients during follow-up, T2 low_150_ (**A**), and T2 low_300_ (**B**). The blue line indicated consistent type 2 low status during follow-up with all values of BEC below 150/300 cells/µL per corresponding threshold and/or FeNO < 25 ppb.

To assess the stability of both T2 low_150_ and T2 low_300_ status, the BEC and FeNO values during follow-up were investigated, Figure 2. Among the T2low_150_ patients (*n* = 31), 20 patients had further BEC and/or FeNO biomarkers measured during follow-up, whereof 11 patients (55%) showed a stable biomarker profile. Correspondingly in T2low_300_ out of 66 patients, 46 had biomarkers measured during follow-up and 33 (72%) showed stable biomarker profile. Some patients with a non-persistent biomarker status had a single higher measure above the threshold (BEC or FeNO), while follow-up measures were again below threshold. Correspondingly, for 11 of 31 (35%) of the patients identified as T2 low_150_, and 20 of 66 (30%) of those identified as T2 low_300_ during baseline, no follow-up measures of BEC and/or FeNO were available, and the biomarker stability could not be assessed.

### 3.4. Exacerbations and Cumulative Corticosteroid Purchases

**Figure 3:**
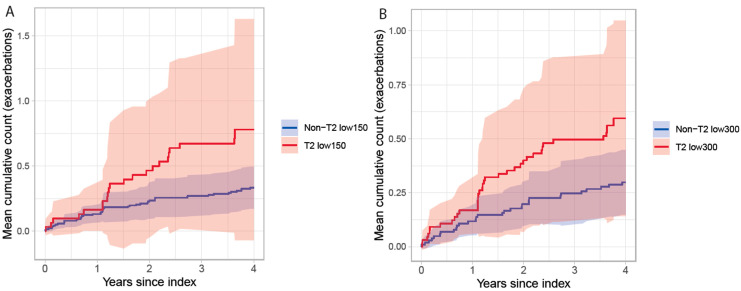
Mean cumulative count of exacerbations requiring hospital care in patients with severe uncontrolled asthma stratified by T2 status for T2low_150_ vs. non-T2 low_150_ (**A**) and T2low_300_ vs. non-T2 low_300_ (**B**).

The proportions of patients with exacerbations requiring hospital care during the baseline period and follow-up in different T2 groups are presented in Table 2. Most patients had no exacerbations requiring hospital care, and the proportion with 1 or more exacerbation was similar between the T2 low and non-T2 low groups during baseline and follow-up. During follow-up, the mean event rate of exacerbations requiring hospital care per 100 patient-years was 19.7 [95% CI: 4.3–45.6] in T2 low_150_ vs. 8.4 [4.7–13.0] in non-T2 low_150_, and 15.0 [5.2–28.9] in T2 low_300_ vs. 7.5 [4.2–11.8] in non-T2 low_300._ Assessing the mean cumulative count of exacerbations per patient during follow-up, no differences were observed between T2 low and non-T2low patients with either stratification, Figure 3.

**Figure 4:**
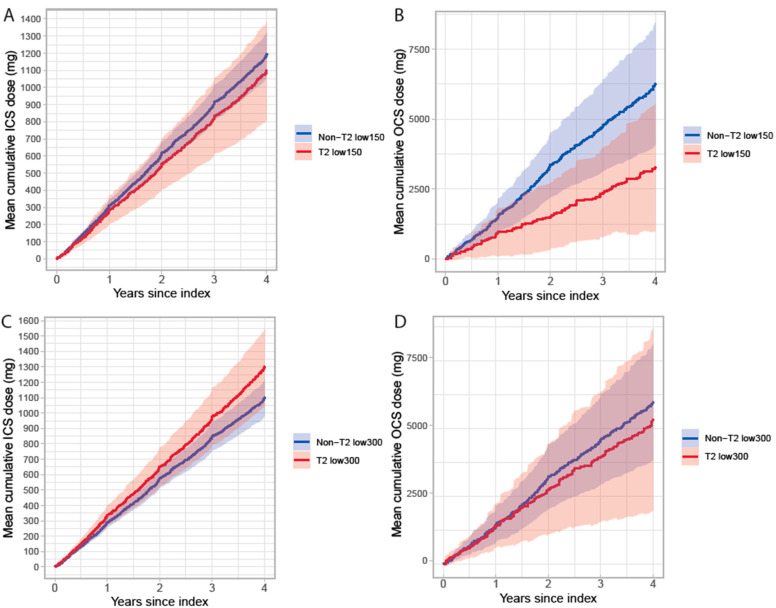
Mean cumulative dose of ICS (**A**,**C**) and OCS (**B**,**D**) during follow-up in patients with severe uncontrolled asthma stratified by T2 status and BEC cut-off 150 (**A**,**B**) and 300 cells/µL (**C**,**D**).

Exacerbations and treatment with corticosteroids may differ by type 2 status. Hence, the cumulative OCS and ICS doses during follow-up were assessed. In the T2 low_300_ vs. non-T2 low_300_ groups patients used OCS similarly (mean cumulative dose at 4 years: 5335 mg [95% CI: 1972, 8697] vs. 5970 mg [3816, 8124], respectively) whereas T2low_150_ purchased only half the amount of OCS during follow-up compared to non-T2low_150_ (mean cumulative dose at 4 years: 3272 mg [95% CI: 978, 5565] vs. 6260 mg [4065, 8453], respectively), Figure 4. The mean cumulative ICS dose did not differ between the groups during follow-up.

## 4. Discussion

This is one of the first studies evaluating the proportion of patients with and the stability of type 2 low biomarker status, during a 4-year follow-up, in a contemporary severe asthma population treated at specialty care with data available from electronic medical records. Among patients with severe uncontrolled asthma, the applied type 2 low biomarker criteria identified type 2 low signature in 18% using BEC cut off < 150 cells/µL + FeNO < 25 ppb, and in 39% of patients using BEC < 300 cells/µL + FeNO < 25 ppb. Novel findings show that there was a difference in biomarker status stability of 72% and 55% between the different BEC cut offs (300 and 150 cells/µL) among patients with severe uncontrolled asthma. Both BEC thresholds identified patients lacking features associated with type 2 high endotype, for instance nasal polyposis, and showed lower IgE concentrations [19]. Choosing low FeNO and BEC <300 cells/µL cut-off seems to select a more stable profile. However, more studies are needed to identify the optimal cut off point for BEC to define a type 2 low patient population.

Of the patients identified as T2low_150_ or T2 low_300_ during baseline but presenting with an unstable biomarker status during follow-up, single biomarker values above the threshold were observed. Of significance is that asthma in these patients was uncontrolled, despite high dose ICS and second controller use and low BEC and FeNO, and the biomarkers remained below the threshold in most patients. To our knowledge no studies have investigated the stability of type 2 inflammatory biomarkers over several years in a similar context to our study i.e., severe uncontrolled type 2 low asthma. A prospective study used S-IgE and BEC in their definition and focused on type 2 high asthma stability over one year in patients with mixed asthma severity [12]. In that study, the stability of type 2 low asthma seemed comparable to our T2 low_300_ group.

A consensus statement proposed an algorithm based on BEC (300/150 cell/µL), FeNO (<25 ppb), and lack of nasal polyps and no anti-IL5 treatment to identify likely non-eosinophilic asthma, but the stability of the included features was not assessed [6]. Repeated or single sputum eosinophils have been used to identify non-eosinophilic asthma, present in approximately 24–50% of patients with untreated or mild to-moderate asthma, but severe uncontrolled asthma patients were not included [2,20,21]. In our study, markers of T2 inflammation, such as sputum eosinophils or serum levels of T2 cytokines, were not available, as they are not routinely measured in clinical practice. Among patients with severe asthma, the prevalence of type 2 low asthma has varied between 9–34% [7,8,22], slightly lower than the detected 18–39% in our study among those with severe uncontrolled asthma.

Most likely within the severe uncontrolled patients we identified as type 2 low, there was a heterogeneity of molecular subgroups, possibly having an impact on the biomarker stability and clinical presentation [23]. One prospective follow-up study noted that among type 2 low patients, identified by FeNO < 20 ppb and BEC < 150 cells/µL, the low biomarker profile persisted during the first exacerbation in 35% of patients. Exacerbations occurred in 50% of type 2 low patients, and at similar rates compared to type 2 high patients, even if the design and definition for type 2 low and exacerbations differed from our study [24]. In addition, asthma symptoms and quality of life have recently been shown to be similar between type 2 low and type 2 high patients [22]. In our study, exacerbations measured as events requiring hospital care were equally frequent in T2 low and non-T2 low patients across both BEC cut offs. For the definition of exacerbations, only those requiring hospital in- or out-patient care could be evaluated. Exacerbations requiring OCS courses could not be assessed as typical purchases included the amount for several OCS courses and were likely kept and used on demand. However, it may be speculated that the T2 low patients may not benefit from OCS [1,20,24], and that hospital visits may be a good indicator of poor asthma control and severe disease. This is also suggested by the finding that the cumulative purchase of OCS of the T2low_150_ group was only about half of that in the non-T2low_150_ group. Clinically, it would be important to thoroughly evaluate whether T2 low patients benefit from OCS-courses to avoid excess steroid use. Further, patients should be evaluated for the benefits of currently available treatment options: adding LAMA to ICS-LABA and considering the dose of ICS as there may be no benefit from a high dose. Moreover, new drugs are developed for type 2 low patients [1,2].

Our study focused on severe uncontrolled asthma and type 2 status. By definition, the patients met the criteria for severe disease based on high dose ICS use. Presumably, the T2 low patients are not as responsive to corticosteroids, which could lead to lower doses of ICS in use and hence underrepresentation of severe T2 low patients in the study. On the other hand, corticosteroids, especially systemic corticosteroids, suppress eosinophils and this may also affect biomarker status. As the BEC values in our study were taken during routine clinical practice, and in-hospital use of OCS was not available for the study, the possible use of OCS during BEC measurements could not be evaluated. To minimize the influence of possible corticosteroid use on BEC and evaluation of T2 status, and to increase validity, at least two baseline measures and additionally one FeNO measure during baseline were required for the T2 low status. In the UK severe asthma registry, over half of type 2 low patients were on maintenance OCS and many of them had earlier high values of BEC [8]; therefore, the evaluation on type 2 low status in patients on treatment is never simple and requires multiple longitudinal measurements [25]. Our analyses utilized biomarkers that are easily available in routine clinical practice, however, prospective studies evaluating these biomarkers are needed to validate the criteria and outcomes presented here.

This study has a few limitations. The included patients likely overrepresent those with severe asthma as the biomarker data were available only from specialty care required for the definition of the type 2 status. Correspondingly, baseline biomarker data to define T2 low status were available for 27% (169 of 637) of the severe uncontrolled patients, with a possibility for a selection bias, likely towards more severe patients with more frequent visits. T2 low status was defined based on the absence of T2 biomarkers, reflecting the current clinical practice to determine T2 status. Prospective studies investigating other probable non-T2 biomarkers such as IL-17, tumor necrosis factor, and type I interferons for the identification of non-T2 status are highly warranted [1,2]. Other limitations come from real-world clinical practice, where all data are not available for all patients and variables can be incomplete or partially missing. In addition, the measures used to assess the T2 low persistency were not repeatedly taken for all patients and the usability of other potential biomarkers could not be evaluated in this setting.

## 5. Conclusions

To conclude, utilizing biomarkers widely available in routine clinical practice, we show that the T2 low biomarker status in severe uncontrolled asthma was stable during follow-up in three quarters of patients with BEC below 300 cells/µL. In addition, both T2 low and non-T2 low groups showed similar exacerbation rates requiring hospital care. Monitoring of inflammatory markers for the evaluation of T2 status with repeated measures is important to ensure adequate and suitable treatment for patients with T2 low asthma.

## Figures and Tables

**Table 1:** Baseline characteristics of severe uncontrolled patients with T2 low and non-T2 low asthma.

		Severe Uncontrolled Asthma			
		T2 Low_150_ *n* = 31	Non-T2 Low_150_ *n* = 138	T2 Low_300_ *n* = 66	Non-T2 Low_300_ *n* = 103
Sex	Male	5 (16%)	45 (33%)	11 (17%)	39 (38%) †
Age at index	Median (IQR)	46 (39, 52)	54 (43, 64) *	48 (39, 55)	56 (46, 66) **
Years since asthma reimbursement start	Median (IQR)	10 (8,15)	13 (4,12)	10 (5,15)	14 (5,22)
	Missing *n*, %	<5 (16.1%)	<5 (<3.6%)	<5 (7.6%)	<5 (<4.9%)
Comorbidities	COPD	<5 (<16.1%)	13 (9.4%)	8 (12%)	8 (7.8%)
	Nasal polyps	0 (0%)	14 (10%)	0 (0%)	14 (14%) *
Smoking	Smoking (ever)	<5 (<16.1%)	16 (12%)	6 (10%)	14 (14%)
	Missing *n*, %	<5 (<16.1%)	7 (5.1%)	6 (9.1%)	<5 (<4.9%)
BMI kg/m^2^	<18.5	<5 (<16.1%)	<5 (<3.6%)	<5 (<7.6%)	<5 (<4.9%)
	>=18.5 to <25	13 (46%)	30 (23%)	22 (37%)	21 (21%)
	>=25 to < 30	7 (25%)	45 (34%)	13 (22%)	39 (39%)
	30>=	7 (25%)	54 (41%)	24 (40%)	37 (37%)
	Missing *n*, %	<5 (<16.1%)	6 (4.3%)	6 (9.1%)	<5 (<4.9%)
BEC cells/µL (maximum)	Median (IQR)	100 (70, 130)	425 (260, 872) **	165 (100, 230)	540 (375, 954) **
Neutrophils cells/µl	Median (IQR)	3.3 (2.1, 5.4)	4.0 (3.1, 5.9)	3.6 (2.6, 5.3)	4.3 (3.1, 6.0)
	Missing *n*, %	<5 (<16.1%)	6 (4.3%)	5 (7.6%)	<5 (<4.9%)
FeNO, ppb (maximum)	Median (IQR)	11 (8, 15)	20 (12, 36) **	12 (9, 17)	24 (14, 50) **
IgE kU/l (maximum)	Median (IQR)	21 (10, 59)	133 (25, 363)	24 (10, 77)	222 (53, 427) **
	Missing *n*, %	6 (19.4%)	33 (23.9%)	16 (24.2%)	23 (22.3%)
Asthma control test (minimum)	Mean (SD)	12.0 (3.3)	11.6 (4.2)	11.1 (3.6)	12.0 (4.3)
	Missing *n*, %	0 (0%)	<5 (<3.6%)	<5 (<7.6%)	<5 (<4.9%)
FEV1 Z-score ^1^	Median (IQR)	1.03 (0.11, 1.75)	0.01 (−1.12, 1.08) *	0.57 (−0.56, 1.53)	−0.01 (−1.16, 0.89) †
	Missing *n*, %	<5 (<16.1%)	7 (5.1%)	7 (10.6%)	<5 (<4.9%)
FVC Z-score ^1^	Median (IQR)	0.93 (−0.28, 1.68)	0.44 (−0.47, 1.24)	0.70 (−0.47, 1.49)	0.41 (−0.45, 1.19)
	Missing *n*, %	<5 (<16.1%)	7 (5.1%)	7 (10.6%)	<5 (<4.9%)
FEV1/FVC Z-score ^1^	Median (IQR)	0.80 (0.74, 0.85)	0.73 (0.66, 0.81) **	0.79 (0.74, 0.84)	0.72 (0.66, 0.79) **
	Missing *n*, %	<5 (<16.1%)	6 (4.3%)	6 (9.1%)	<5 (<4.9%)
ICS dose µg/day	Median (IQR), baseline	705 (483, 974)	728 (557, 979)	731 (568, 997)	714 (544, 976)
	Median (IQR), past year	739 (513, 1006)	842 (493, 1068)	801 (462, 1129)	821 (513, 1068)
OCS dose mg/day	Median (IQR), baseline	2.0 (1.3, 3.8)	2.9 (1.5, 6.0)	2.1 (1.3, 5.0)	3.2 (1.7, 6.0)
	Median (IQR), past year	0 (0, 4)	2 (0, 5)	0 (0, 4)	2 (0, 5)
Respiratory antibiotic purchases ^2^	At least one purchase *n*, %	29 (94%)	134 (97%)	62 (94%)	101 (98%)
	Number of purchases Median (IQR)	7 (4, 14)	7 (3, 13)	7 (3, 14)	7 (3, 13)

^1^ latest value before index. ^2^ doxycycline J01AA02, amoxicillin J01CA04, amoxicillin clavulanate J01CR02, azithromycin J01FA10, clarithromycin J01FA09. Z score—how many standard deviations the measured value is from predicted [(observed-predicted)/standard deviation]. All patients had been treated with a second controller [Leukotriene Receptor Antagonists (LTRA), Long-acting beta-agonist (LABA), Long-acting muscarinic antagonists (LAMA)] at baseline, per definition of severe asthma. † *p* < 0.05, * *p* < 0.01; ** *p* < 0.001.

**Table 2:** Exacerbations requiring hospital care during baseline and follow-up in severe uncontrolled patients per T2 low status.

		T2 Low_150_ *n* = 31	Non-T2 Low_150_ *n* = 138	T2 Low_300_ *n* = 66	Non-T2 Low_300_ *n* Same = 103
Baseline period	0	19 (61%)	91 (66%)	42 (64%)	68 (66%)
	1	5 (16%)	25 (18%)	12 (18%)	18 (17%)
	2	0 (0%)	8 (5.8%)	<5 (<7.6%)	6 (5.8%)
	3	<5 (<16.1%)	5 (3.6%)	<5 (<7.6%)	5 (4.9%)
	4+	<5 (<16.1%)	9 (6.5%)	7 (11%)	6 (5.8%)
	One or more	12 (39%)	47 (34%)	24 (36%)	35 (34%)
Follow-up	0	23 (74%)	110 (80%)	51 (77%)	82 (80%)
	1	<5 (<16.1%)	22 (16%)	9 (14%)	17 (17%)
	2	<5 (<16.1%)	<5 (<3.6%)	<5 (<7.6%)	<5 (<4.9%)
	3	0 (0%)	<5 (<3.6%)	0 (0%)	<5 (<4.9%)
	4+	<5 (<16.1%)	<5 (<3.6%)	<5 (<7.6%)	<5 (<4.9%)
	One or more	8 (26%)	28 (20%)	15 (23%)	21 (20%)

No statistical significance (*p* > 0.05) was observed between groups.

## Data Availability

The data that support the findings of this study were collected by following the guidance and application process of Findata, the central permission authority in Finland. Restrictions apply to the data, where only persons named in the study permission have access to the pseudonymized micro data for the current study, and so are not publicly available.

## Supplementary Materials

The following supporting information can be downloaded at: https://www.mdpi.com/article/10.3390/biom13071118/s1, Figure S1: Study design; Figure S2: Number of patients with BEC < 150 cells/µL at least twice and FeNO < 25 ppb during baseline, and the overlap of these criteria for the formation of the T2 low_150_ group (A). Number of patients with BEC < 300 cells/µL at least twice and FeNO < 25 ppb during baseline, and the overlap of these criteria for the formation of the T2 low_300_ group (B). Patients in the T2 low_150_ were included in the T2 low_300_ group.

## Abbreviations

BEC	blood eosinophil count
ER	emergency room visit
FeNO	fractional exhaled nitric oxide
FEV1	forced expiratory volume in one second
FVC	forced vital capacity
IgE	immunoglobulin E
ICS	inhaled corticosteroids
IL	interleukin
LABA	Long-Acting Beta-Agonist
LAMA	Long-Acting Muscarinic Antagonists
LTRA	Leukotriene Receptor Antagonists
Non-T2 low	patients with type 2 inflammatory biomarkers
OCS	oral corticosteroids
T2	type 2 inflammation
T2 low	absence of type 2 inflammatory biomarkers

## References

[1] Kyriakopoulos C., Gogali A., Bartziokas K., Kostikas K. (2021). Identification and Treatment of T2-Low Asthma in the Era of Biologics. ERJ Open Res..

[2] Hinks T.S.C., Levine S.J., Brusselle G.G. (2021). Treatment Options in Type-2 Low Asthma. Eur. Respir. J..

[3] Niessen N.M., Fricker M., McDonald V.M., Gibson P.G. (2022). T2-Low: What Do We Know?. Ann. Allergy Asthma Immunol..

[4] Rupani H., Fong W.C.G., Kyyaly M.A., Kurukulaaratchy R.J. (2021). Recent Insights into the Management of Inflammation in Asthma. J. Inflamm. Res..

[5] Kuruvilla M.E., Lee F.E.-H., Lee G.B. (2019). Understanding Asthma Phenotypes, Endotypes, and Mechanisms of Disease. Clinic. Rev. Allergy Immunol..

[6] Heaney L.G., Perez de Llano L., Al-Ahmad M., Backer V., Busby J., Canonica G.W., Christoff G.C., Cosio B.G., FitzGerald J.M., Heffler E. (2021). Eosinophilic and Noneosinophilic Asthma. Chest.

[7] Denton E., Price D.B., Tran T.N., Canonica G.W., Menzies-Gow A., FitzGerald J.M., Sadatsafavi M., Perez de Llano L., Christoff G., Quinton A. (2021). Cluster Analysis of Inflammatory Biomarker Expression in the International Severe Asthma Registry. J. Allergy Clin. Immunol. Pract..

[8] Jackson D.J., Busby J., Pfeffer P.E., Menzies-Gow A., Brown T., Gore R., Doherty M., Mansur A.H., Message S., Niven R. (2021). Characterisation of Patients with Severe Asthma in the UK Severe Asthma Registry in the Biologic Era. Thorax.

[9] Castro M., Corren J., Pavord I.D., Maspero J., Wenzel S., Rabe K.F., Busse W.W., Ford L., Sher L., FitzGerald J.M. (2018). Dupilumab Efficacy and Safety in Moderate-to-Severe Uncontrolled Asthma. N. Engl. J. Med..

[10] Ortega H.G., Liu M.C., Pavord I.D., Brusselle G.G., FitzGerald J.M., Chetta A., Humbert M., Katz L.E., Keene O.N., Yancey S.W. (2014). Mepolizumab Treatment in Patients with Severe Eosinophilic Asthma. N. Engl. J. Med..

[11] Menzies-Gow A., Corren J., Bourdin A., Chupp G., Israel E., Wechsler M.E., Brightling C.E., Griffiths J.M., Hellqvist Å., Bowen K. (2021). Tezepelumab in Adults and Adolescents with Severe, Uncontrolled Asthma. N. Engl. J. Med..

[12] Maison N., Omony J., Illi S., Thiele D., Skevaki C., Dittrich A.-M., Bahmer T., Rabe K.F., Weckmann M., Happle C. (2022). T2-High Asthma Phenotypes across Lifespan. Eur. Respir. J..

[13] Habib N., Pasha M.A., Tang D.D. (2022). Current Understanding of Asthma Pathogenesis and Biomarkers. Cells.

[14] Ricciardolo F.L.M., Carriero V., Bertolini F. (2021). Which Therapy for Non-Type(T)2/T2-Low Asthma. J. Pers. Med..

[15] Robinson D., Humbert M., Buhl R., Cruz A.A., Inoue H., Korom S., Hanania N.A., Nair P. (2017). Revisiting Type 2-High and Type 2-Low Airway Inflammation in Asthma: Current Knowledge and Therapeutic Implications. Clin. Exp. Allergy.

[16] FINLEX®®-Säädökset Alkuperäisinä: Laki Sosiaali-ja Terveystietojen Toissijaisesta… 552/2019. http://www.finlex.fi/fi/laki/alkup/2019/20190552.

[17] Chung K.F., Wenzel S.E., Brozek J.L., Bush A., Castro M., Sterk P.J., Adcock I.M., Bateman E.D., Bel E.H., Bleecker E.R. (2014). International ERS/ATS Guidelines on Definition, Evaluation and Treatment of Severe Asthma. Eur. Respir. J..

[18] R Core Team (2022). R: A Language and Environment for Statistical Computing.

[19] Maspero J., Adir Y., Al-Ahmad M., Celis-Preciado C.A., Colodenco F.D., Giavina-Bianchi P., Lababidi H., Ledanois O., Mahoub B., Perng D.-W. (2022). Type 2 Inflammation in Asthma and Other Airway Diseases. ERJ Open Res..

[20] Green R.H. (2002). Analysis of Induced Sputum in Adults with Asthma: Identification of Subgroup with Isolated Sputum Neutrophilia and Poor Response to Inhaled Corticosteroids. Thorax.

[21] McGrath K.W., Icitovic N., Boushey H.A., Lazarus S.C., Sutherland E.R., Chinchilli V.M., Fahy J.V. (2012). A Large Subgroup of Mild-to-Moderate Asthma Is Persistently Noneosinophilic. Am. J. Respir. Crit. Care Med..

[22] Frøssing L., Klein D.K., Hvidtfeldt M., Obling N., Telg G., Erjefält J.S., Bodtger U., Porsbjerg C. (2022). Distribution of Type 2 Biomarkers and Association with Severity, Clinical Characteristics and Co-Morbidities in the BREATHE Real-Life Asthma Population. ERJ Open Res..

[23] Kuo C.-H.S., Pavlidis S., Loza M., Baribaud F., Rowe A., Pandis I., Sousa A., Corfield J., Djukanovic R., Lutter R. (2017). T-Helper Cell Type 2 (Th2) and Non-Th2 Molecular Phenotypes of Asthma Using Sputum Transcriptomics in U-BIOPRED. Eur. Respir. J..

[24] McDowell P.J., Busby J., Hanratty C.E., Djukanovic R., Woodcock A., Walker S., Hardman T.C., Arron J.R., Choy D.F., Bradding P. (2022). Exacerbation Profile and Risk Factors in a Type-2–Low Enriched Severe Asthma Cohort: A Clinical Trial to Assess Asthma Exacerbation Phenotypes. Am. J. Respir. Crit. Care Med..

[25] Lugogo N.L., Kreindler J.L., Martin U.J., Cook B., Hirsch I., Trudo F.J. (2020). Blood Eosinophil Count Group Shifts and Kinetics in Severe Eosinophilic Asthma. Ann. Allergy Asthma Immunol..

