# Supplementary material for: Type 2 Low Biomarker Stability and Exacerbations in Severe Uncontrolled Asthma
Source: Biomolecules. 2023 Jul 13;13(7):1118. doi: 10.3390/biom13071118 (PMC10377379; doi:10.3390/biom13071118)
Supplement: Supplementary file 1 [file biomolecules-13-01118-s001.zip › biomolecules-2478108-supplementary.pdf]

Supplementary material

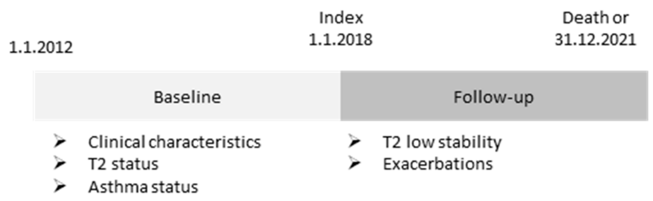

Figure S1. Study design.

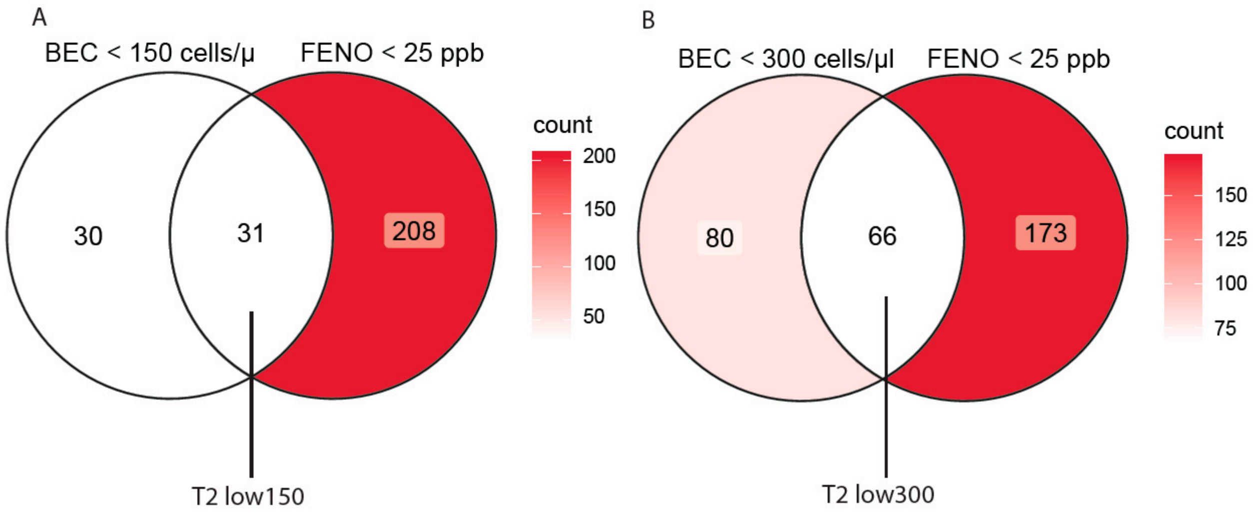

Figure S1. Number of patients with BEC < 150 cells/µl at least twice and FeNO < 25 ppb during baseline, and the overlap of these criteria for the formation of the T2 low_{150} group (A). Number of patients with BEC < 300 cells/µl at least twice and FeNO < 25 ppb during baseline, and the overlap of these criteria for the formation of the T2 low_{300} group (B). Patients in the T2 low_{150} were included in the T2 low_{300} group.
